# Aging Affects Neural Synchronization to Speech-Related Acoustic Modulations

**DOI:** 10.3389/fnagi.2016.00133

**Published:** 2016-06-15

**Authors:** Tine Goossens, Charlotte Vercammen, Jan Wouters, Astrid van Wieringen

**Affiliations:** Research Group Experimental Oto-rhino-laryngology (ExpORL), Department of Neurosciences, KU Leuven - University of LeuvenLeuven, Belgium

**Keywords:** aging, amplitude modulations, auditory steady-state response (ASSR), hemispheric lateralization, neural oscillations, synchronization, temporal processing

## Abstract

As people age, speech perception problems become highly prevalent, especially in noisy situations. In addition to peripheral hearing and cognition, temporal processing plays a key role in speech perception. Temporal processing of speech features is mediated by synchronized activity of neural oscillations in the central auditory system. Previous studies indicate that both the degree and hemispheric lateralization of synchronized neural activity relate to speech perception performance. Based on these results, we hypothesize that impaired speech perception in older persons may, in part, originate from deviances in neural synchronization. In this study, auditory steady-state responses that reflect synchronized activity of theta, beta, low and high gamma oscillations (i.e., 4, 20, 40, and 80 Hz ASSR, respectively) were recorded in young, middle-aged, and older persons. As all participants had normal audiometric thresholds and were screened for (mild) cognitive impairment, differences in synchronized neural activity across the three age groups were likely to be attributed to age. Our data yield novel findings regarding theta and high gamma oscillations in the aging auditory system. At an older age, synchronized activity of theta oscillations is increased, whereas high gamma synchronization is decreased. In contrast to young persons who exhibit a right hemispheric dominance for processing of high gamma range modulations, older adults show a symmetrical processing pattern. These age-related changes in neural synchronization may very well underlie the speech perception problems in aging persons.

## Introduction

With advancing age, people experience greater difficulty following conversations, especially in the presence of background noise and/or when multiple speakers are talking (Pichora-Fuller and Souza, 2003). This impaired speech perception can be attributed to age-related peripheral hearing loss (Abel et al., 2000) as well as to declining cognitive abilities (Van der Linden et al., 1999) and changes in central temporal processing (Martin and Jerger, 2005).

With regard to temporal processing, it has been shown that neural oscillations in the central auditory system synchronize their phase patterns to temporal features of speech sounds (Ahissar et al., 2001; Luo and Poeppel, 2007; Cogan and Poeppel, 2011). Neural oscillations are classified in five frequency ranges, i.e., delta (< 4 Hz), theta (~4–8 Hz), alpha (~8–13 Hz), beta (~13–30 Hz), and gamma (> 30 Hz).

The synchronized activity of oscillations can be investigated electrophysiologically by means of the auditory steady-state response (ASSR) (for a review on the ASSR see Picton, 2010). The size of the ASSR reflects the degree to which neural oscillatory activity synchronizes to acoustic modulations that coincide with the characteristic frequency of the oscillations. For instance, the size of the 40 Hz ASSR reflects the degree of synchronized activity of low gamma oscillations in response to 40 Hz acoustic modulations.

Taken together, aging goes together with difficulties in speech perception, which, in turn, is mediated by synchronized neural activity. Therefore, we assume that ASSRs may reveal age-related changes in neural synchronization to modulations that are important for speech perception.

Numerous studies have investigated temporal features of speech and have demonstrated that the speech envelope, referring to the slowly varying acoustic amplitude of continuous speech, plays a key role in speech perception (e.g., Shannon et al., 1995; Ahissar et al., 2001). This results from the speech envelope conveying both prosodic and linguistic information that is crucial for accurate speech perception (Rosen, 1992; Drullman et al., 1994; Peelle and Davis, 2012). Interestingly, the speech envelope is characterized by theta and beta range modulations. The importance of synchronized activity of these low-frequency oscillations, and theta oscillations in particular, has been demonstrated in research on speech intelligibility (Ahissar et al., 2001; Luo and Poeppel, 2007; Doelling et al., 2014), speech discrimination (Ortiz-Mantilla et al., 2013), and speech stream segregation (Kerlin et al., 2010).

Associations between theta synchronization and speech perception likely result from the theta frequency range coinciding with the rate of occurrence of syllables in continuous speech (~4 Hz, 250 ms) (Plomp, 1983; Steeneken and Houtgast, 1983). After all, syllables are critical speech units, driving speech perception (Greenberg et al., 2003; Poeppel, 2003). Furthermore, it has been demonstrated that theta synchronization mediates the parsing of continuous speech into syllable-sized chunks (e.g., Doelling et al., 2014).

Remarkably, auditory research on potential age-related changes in synchronized activity of low-frequency oscillations is scarce. This paucity is in sharp contrast with the large body of such research in the cognitive domain. A variety of cognitive studies have demonstrated substantial dissimilarities between young and older adults in low-frequency neural synchronization, which, moreover, seemed to underlie age-related differences in memory performance (e.g., Karrasch et al., 2004; Cummins and Finnigan, 2007; Kober et al., 2016). To the best of our knowledge, there is only one study in the auditory domain in which the degree of synchronized theta activity is compared across different age groups (Tlumak et al., 2015). In this study, 5 Hz ASSRs were recorded in young, middle-aged, and older adults. The ASSR appeared to be larger in the older compared to the young group, suggesting increased theta synchronization with advancing age. In addition, two studies have investigated low-frequency beta synchronization in different age groups by means of the 20 Hz ASSR (Leigh-Paffenroth and Fowler, 2006; Tlumak et al., 2015). Both studies indicated that there were no age-related changes in the synchronized activity of beta oscillations.

Similar to theta and beta oscillations, high-frequency gamma oscillations are associated with speech perception. More specifically, amplitude modulations in the gamma range make up the speech feature “periodicity” (50–500 Hz), which reflects the fundamental frequency of speech. The periodicity yields crucial information about voicing and stress and, thereby, contributes to speech intelligibility (Rosen, 1992). Interestingly, evidence exists that reduced neural encoding of gamma range acoustic modulations relates to impaired speech perception (Dimitrijevic et al., 2001, 2004; Leigh-Paffenroth and Fowler, 2006; Anderson et al., 2011; Manju et al., 2014). A number of studies have examined whether high-frequency ASSRs change with age. At the lower end of the gamma range (~40 Hz), no age-related differences have been observed (Muchnik et al., 1993; Boettcher et al., 2001; Purcell et al., 2004; Leigh-Paffenroth and Fowler, 2006; Grose et al., 2009), whereas for high gamma frequencies (≥ 80 Hz), ASSRs seem to decrease with advancing age, suggesting an age-related decline in synchronized activity of high gamma oscillations (Dimitrijevic et al., 2004; Purcell et al., 2004; Leigh-Paffenroth and Fowler, 2006; Grose et al., 2009; Parthasarathy et al., 2010).

In addition to the degree of synchronized neural activity, a growing number of studies is investigating the importance of hemispheric lateralized activity for speech perception. Hemispheric lateralization refers to an asymmetry in synchronized neural activity with a higher degree of synchronization over one hemisphere compared to the other. Such hemispheric asymmetry likely suggests functional specialization of that hemisphere for the temporal feature being processed. With regard to the speech envelope, a right hemispheric (RH) lateralization is suggested for theta range modulations (~4 Hz), whereas the left hemisphere (LH) preferentially extracts beta range modulations (~20 Hz) (e.g., Poeppel, 2003; Boemio et al., 2005; Liem et al., 2014). For gamma range modulations, a RH dominance has been demonstrated for both low (~40 Hz; Ross et al., 2005) and high gamma frequencies (≥ 80 Hz; Schoonhoven et al., 2003; Hertrich et al., 2004).

Recent studies have suggested that impaired speech processing in persons with dyslexia may have its origin in reduced hemispheric lateralization for processing of speech-related acoustic modulations (Goswami, 2011; Hämäläinen et al., 2012). Also, advancing age appears to be associated with deviances in hemispheric asymmetries for high-level auditory and cognitive processing (e.g., detecting morphosyntactic anomalies and memory retrieval) (Greenwald and Jerger, 2001; Cabeza, 2002; Berlingeri et al., 2013). Up to date, however, there is a lack of research concerning potential effects of age on hemispheric lateralization for low-level auditory processing, like neural encoding of speech-related acoustic modulations.

The objective of this study was to determine whether aging affects the degree and/or lateralization of synchronized theta, beta, and gamma activity in response to speech-related acoustic modulations.

## Materials and methods

### Participants

In this study, we included three narrow age groups: 20–30, 50–60, and 70–80 years of age. Age group characteristics are presented in Table 1.

**Table 1:** **Age group characteristics**.

	**Young**	**Middle-aged**	**Older**
Number	19	20	14
Women/Men	10/9	10/10	10/4
Mean age ± SD	22 ± 1	53 ± 2	74 ± 3

Number of participants, number of women/men, mean age ± SD (years).

**Figure 1:**
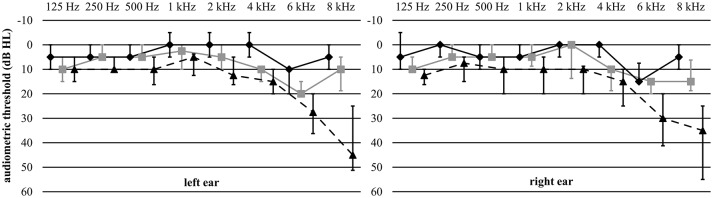
**Audiometric thresholds**. Median audiometric thresholds (dB HL) of the young, middle-aged, and older participants are represented by the black, gray, and dashed lines, respectively. Error bars indicate the interquartile range.

All subjects were Dutch native speakers and were carefully selected through intensive screening across Flanders (Belgium). Importantly, participants had normal hearing in both ears: audiometric thresholds were ≤ 25 dB HL at all octave frequencies from 125 Hz up to and including 4 kHz (see Figure 1). Their hearing was considered symmetrical based on criteria derived from the AMCLASS algorithm (Margolis and Saly, 2008). This stringent auditory criterion was indispensable in order to prevent differences in peripheral hearing from confounding the results (e.g., Picton et al., 2003). We administered a short interview to assure that participants had no medical history of brain injury, neurological disorders, or tinnitus. They were right-handed as assessed by the Edinburgh Handedness Inventory (Oldfield, 1971) and were screened for mild cognitive impairment by means of the Montreal Cognitive Assessment Task, i.e., all subjects scored ≥ 26/30 (Nasreddine et al., 2005). It has been proven that this cut-off score yields excellent sensitivity for detecting mild cognitive impairments. As such, the cognitive screening ensured that all participants had cognitive capacities within the normal range. For the scope of this study, it was required that participants had no indication of (mild) cognitive impairment as cognitive problems are known to affect synchronized activity of neural oscillations (Huang et al., 2000; Osipova et al., 2006; van Deursen et al., 2011).

Taken together, these strict selection criteria ensured that differences in neural synchronization across the three age groups were likely to be attributed to age. They did impede the recruitment of participants, and older participants in particular. Only 14 out of 225 older candidates (6%) screened could be retained for further testing. This low rate was not unexpected as only 10% of men and 50% of women aged 70 years and older have thresholds ≤ 25 dB HL up to and including 4 kHz (International Organization for Standardization, 2000). Furthermore, from the age of 70 onwards, there is a steep decrease in the proportion of persons having cognitive abilities within the normal range, going from 82% to 52% between the ages of 70 and 80 (Yesavage et al., 2002).

This study was approved by the Medical Ethical Committee of the University Hospitals and University of Leuven (approval number B322201214866). After fully informing the participants about the study, they gave their written informed consent.

### Auditory stimulation

The stimulation set-up was controlled by a laptop running a software platform that was developed at our lab. Octave bands of white noise centered at 1 kHz were presented to the participants with ER-3A insert phones. The noise, which was generated in Matlab R2011b (The Mathworks Inc., Natick, MA, USA, 2011), was representative of speech since important cues to speech identification are centered around 1 kHz (e.g., vowel formants; Peterson and Barney, 1952). The noise was 100% amplitude modulated at approximately 4, 20, 40, and 80 Hz, representing theta, beta, low gamma and high gamma frequencies, respectively. More specifically, we applied frequencies near 4, 20, 40, and 80 Hz (i.e., 3.91, 19.53, 40.04, and 80.08 Hz) to prevent electromagnetic artifacts, occurring at integer frequencies, from affecting ASSR detection (e.g., line noise at 50 Hz). Every modulation frequency was presented in three modalities: to the left ear (L), to the right ear (R), and bilaterally, diotically to both ears (BI). This enabled us to investigate whether effects of age on the ASSRs interacted with the side of stimulation.

Stimulus intensity was set to 70 dB SPL. Calibration was performed with a Brüel and Kjær type 2260 sound level meter, a type 4189 half-inch microphone and a 2 cc coupler. An RME Hammerfall DSP Multiface II soundcard was connected to the laptop by a PCMCIA HDSP Cardbus, converting the stimulus at a sampling rate of 32 kHz.

Each of the 12 stimulus conditions (4 frequencies × 3 sides of stimulation) went on for 300 s. The order of conditions was randomized among participants.

### EEG recording

EEG recordings were made in a double-walled soundproof booth with Faraday cage. Participants lay down on a bed and watched a silent movie with subtitles. This passive listening paradigm was adopted from previous studies (e.g., Tremblay et al., 2002, 2003; Anderson et al., 2011; Ruggles et al., 2011). It was important that none of the participants paid attention to the auditory stimuli because attention affects neural processing (Sussman et al., 2002; Bharadwaj et al., 2014). Watching a movie also prevented subjects from falling asleep which otherwise might have affected the cortically generated neural responses, i.e., the 4 Hz and 20 Hz ASSR (Cohen et al., 1991; Purcell et al., 2004). Several imaging studies have indicated that these low-frequency ASSRs have their main neural generators in cortical regions (Giraud et al., 2000; Herdman et al., 2002; Schönwiesner and Zatorre, 2009). The subjects were encouraged to lie quietly and relaxed during auditory stimulation to prevent movements and muscle tensions from distorting the EEG signal.

**Figure 2:**
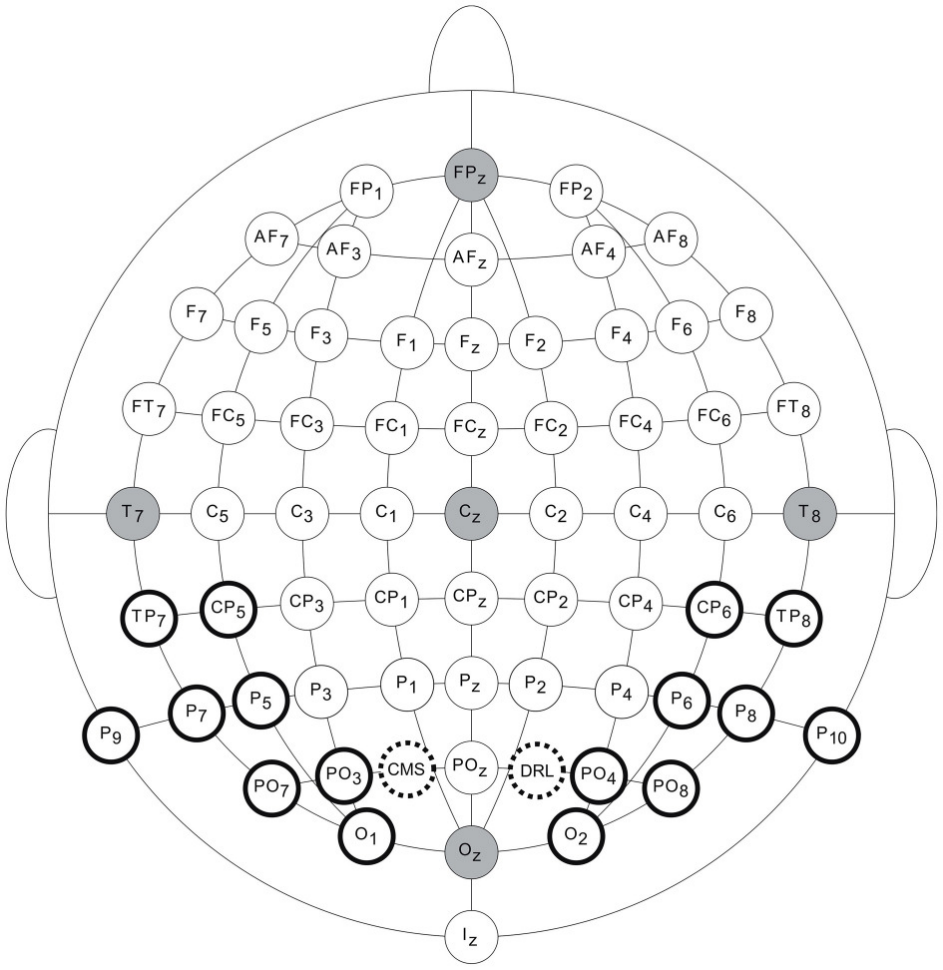
**Electrode configuration and selection**. 64 active electrodes placed according to the 10-10 electrode system and two complementary electrodes, CMS and DRL. The electrodes that were selected for further analyses, are encircled in black.

EEG signals were recorded with the ActiveTwo system of BioSemi (BioSemi B.V., Amsterdam, the Netherlands, 2010), using 64 active Ag/AgCl electrodes. The array of 64 active electrodes was complemented by two electrodes, CMS and DRL, serving as the common electrode and current return path, respectively. The electrodes were mounted in head caps containing electrode holders according to the 10-10 electrode system (American Clinical Neurophysiology Society, 2006) (see Figure 2). An electrolyte gel was injected in the electrode holders to achieve high scalp-electrode conductivity. Electrode offsets, i.e., a running average of the voltage measured between the common electrode and each active electrode, were checked after the participant was laid on the bed and they were continuously monitored during the measurements. If electrode offsets exceeded a voltage of ± 30 mV, a small amount of electrolyte gel was added to lower the offsets, thereby ensuring good quality EEG signals.

The recordings were digitized by an A/D box at a sampling rate of 8192 Hz with a gain of 32.25 nV/bit and sent to a USB 2.0 interface. The recorded EEG signal was displayed on screen and stored on hard disk using ActiView software (BioSemi B.V., Amsterdam, the Netherlands, 2010).

### EEG processing

**Figure 3:**
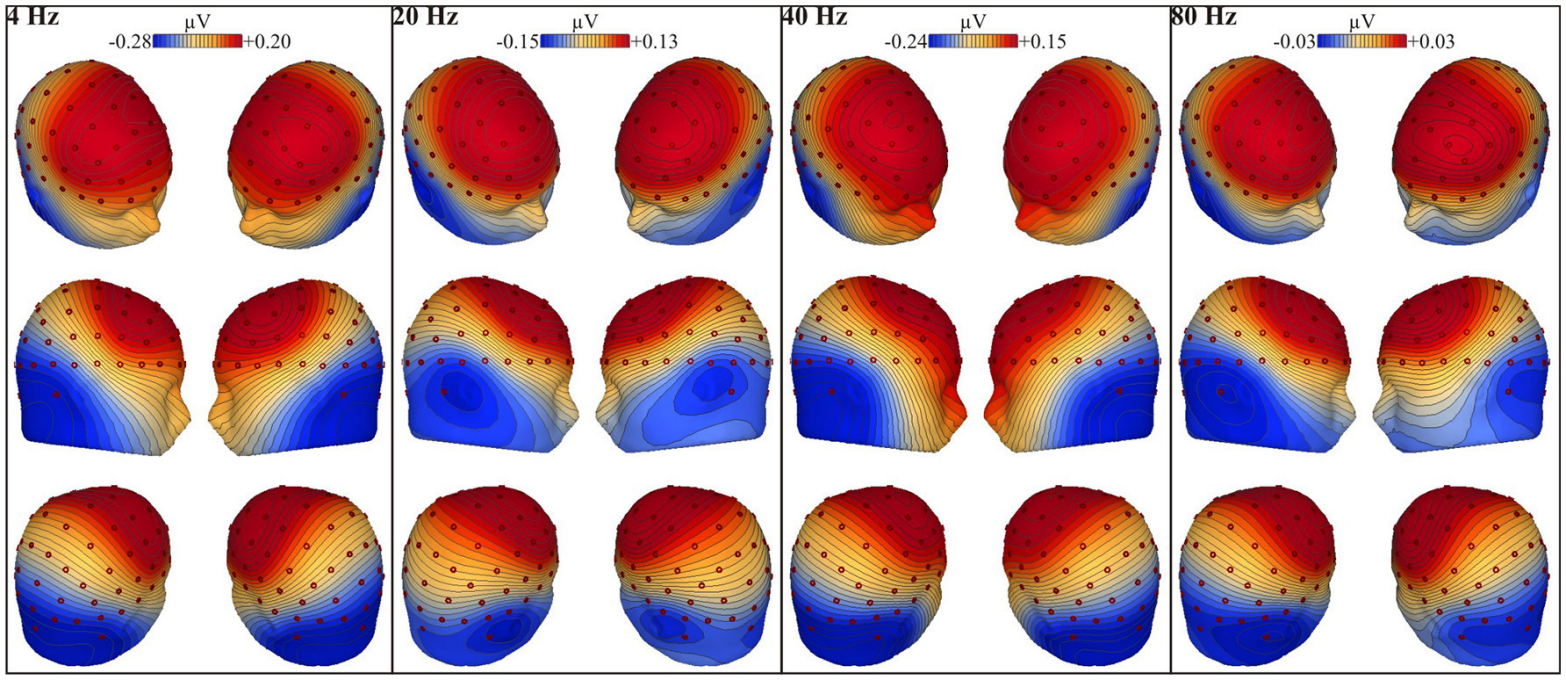
**Whole-head voltage topographic maps**. The EEG voltage maps are plotted with reference-free interpolation and show the scalp distribution of the instantaneous voltage at the maximum positive peak of the grand mean averaged response period. The 4, 20, 40, and 80 Hz grand mean response period is obtained by averaging 256 artifact-free epochs from all participants (*N* = 53) for the three sides of stimulation (L, R, BI). The panels display the EEG voltage topography for the 4, 20, 40, and 80 Hz modulation frequencies (from left to right panels, respectively) from six different viewpoints. Red buttons represent the electrode locations. The voltage distributions are plotted on a color shade minimum-maximum scale going from blue (negative polarity) to red (positive polarity), in which a darker tint reflects a higher voltage value.

**Figure 4:**
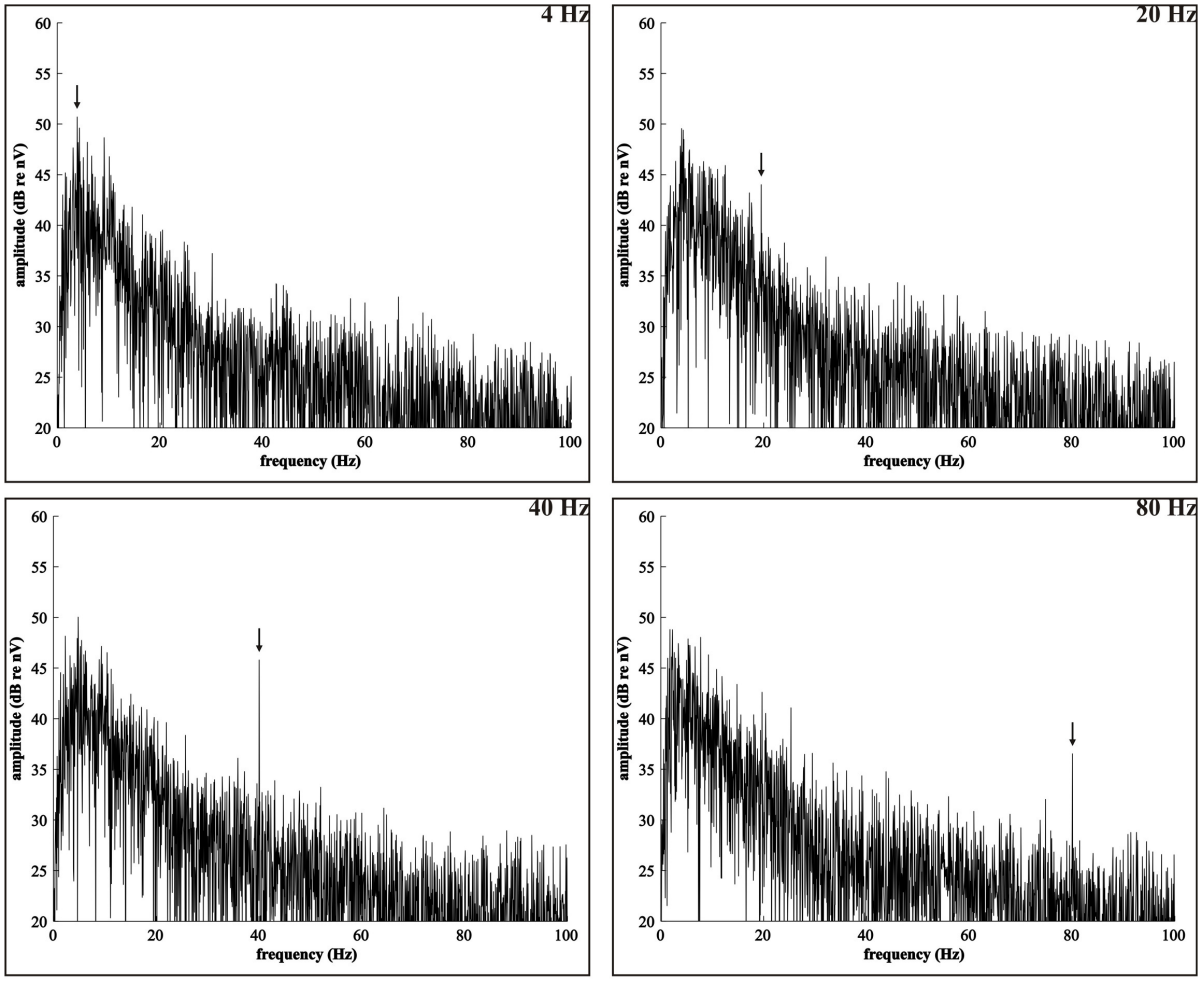
**FFT spectra**. EEG frequency spectra of a young participant, obtained by the procedure outlined in Section EEG Processing. The spectra represent the neural responses to the 4, 20, 40, and 80 Hz acoustic modulations that were recorded over the electrode selection in the right hemisphere (see Figure 2), when presenting the stimuli to the right ear. Spectral amplitudes are converted to dB by applying a logarithmic transformation with reference to 1 nV. The arrows indicate the 4, 20, 40, and 80 Hz ASSR, i.e., the spectral peak in the respective modulation frequency bin that stands out above the adjacent frequency bins which contain EEG noise, induced by the auditory stimulus.

Based on triggers, sent from the stimulation to the recording set-up to synchronize the acoustic stimulation and EEG recording, the EEG recording was segmented into epochs of 1.024 s, each including an integer number of 4, 20, 40, or 80 Hz modulation cycles (John and Picton, 2000b). This resulted in 292 epochs per recording (300 s/1.024 s). We implemented a zero phase high-pass filter with a cut-off frequency of 2 Hz and a slope of 12 dB per octave to exclude distortions caused by skin potentials and the DC component of the amplifier. Artifact rejection was applied with BESA Research 5.3 software (BESA GmbH, Gräfelfing, Germany, 2010). Epochs in which the amplitude exceeded 100 μV were rejected. If fewer than 256 epochs were considered artifact-free, the amplitude rejection level was gradually increased in steps of 10 μV up to and including 150 μV. The first 256 artifact-free epochs were imported into Matlab R2011b (The Mathworks Inc., Natick, MA, USA, 2011) and referenced to Cz since this vertex electrode optimally fits the coronal orientation of auditory current dipoles (e.g., Scherg et al., 1989; Picton et al., 1999) (see Figure 3). This series of epochs was chunked into four sweeps of 64 consecutive epochs. No windowing procedure was applied. The sweeps were averaged in the time domain, thereby reducing the noise in the EEG signal (Dobie and Wilson, 1996; John et al., 1998). Then, a transformation into the frequency domain was performed by a Fast Fourier Transformation (FFT). As the averaged sweep ranged a time interval of 65.5 s (64 × 1.024 s), the FFT spectrum had a high frequency resolution, i.e., 0.015 Hz. Finally, the inverse gain of the high-pass filter was added to the spectral power to compensate for the filter attenuation. Figure 4 shows the resulting FFT spectra of a randomly selected participant.

### ASSR evaluation

#### Testing for significance

We applied an *F* test to verify the presence of an ASSR. The *F* test compared the power in the modulation frequency bin to the power in 120 adjacent bins, i.e., 60 bins below and 60 bins above the modulation frequency bin, each corresponding to a frequency range of 0.9 Hz (60 × 0.015 Hz). This resulted in an *F* ratio with 2 and 240 degrees of freedom. As such, an ASSR was judged as significantly different from the EEG noise when the signal-to-noise ratio (SNR) was 4.8 dB (*p* < 0.05) (Dobie and Wilson, 1996; John and Picton, 2000a; Picton et al., 2005).

#### Electrode selection

**Figure 5:**
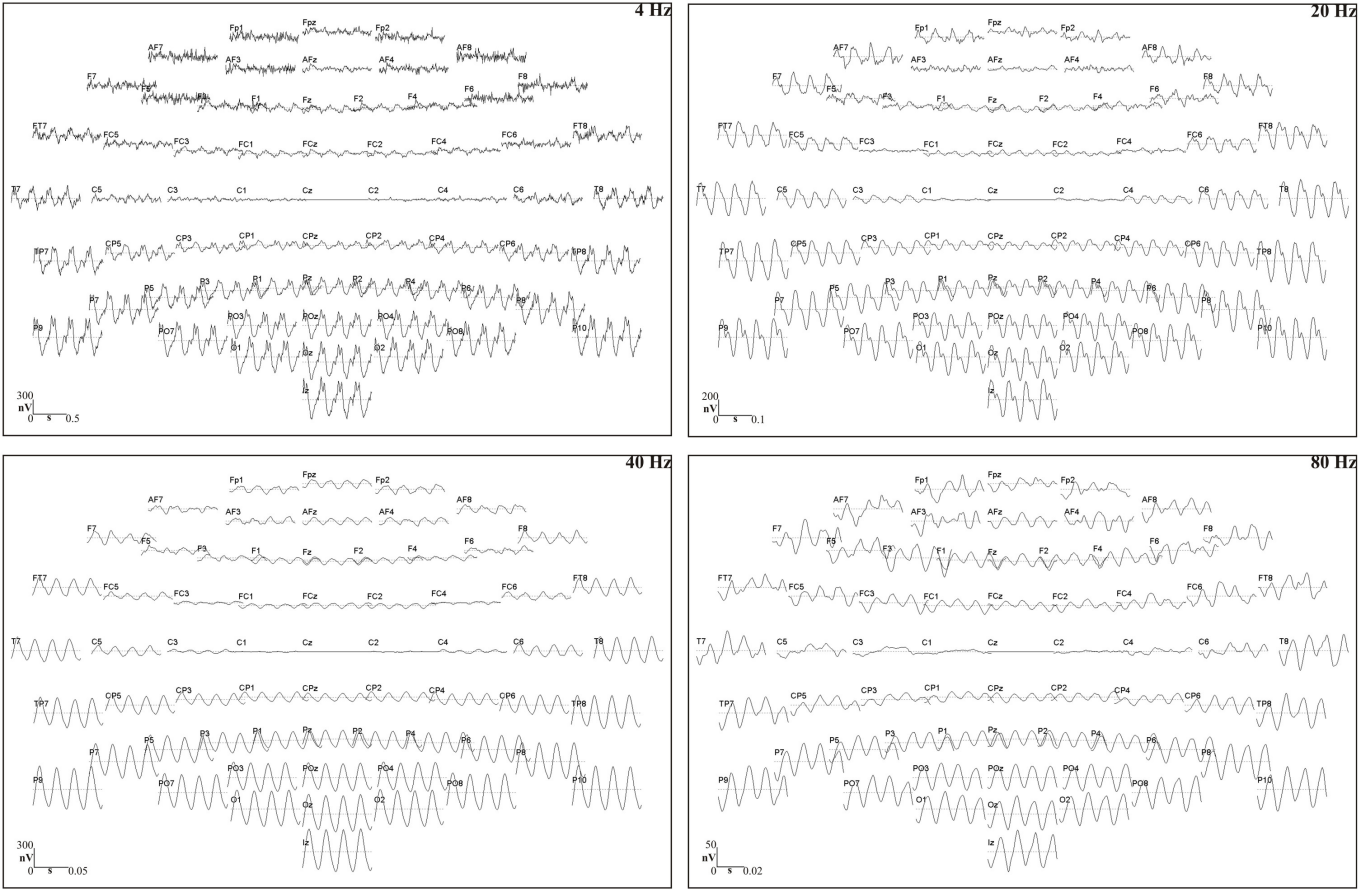
**Auditory evoked potentials topographic maps**. The evoked potentials are referenced to the vertex electrode (Cz) and represent the time-domain grand mean averaged response to four cycles of the 4, 20, 40, or 80 Hz modulated noise. The 4, 20, 40, and 80 Hz grand mean response period is obtained by averaging 256 artifact-free epochs from all participants (*N* = 53) for the three sides of stimulation (L, R, BI). The waveforms are plotted at the approximate electrode locations viewed from the top of the head with the nose at the top of the figure.

For the ASSR evaluation, only neural responses recorded by electrodes that were selected as follows, were analyzed. In order to examine hemispheric lateralization, we excluded all midline electrodes and included pairs of electrodes, mirrored across hemispheres (e.g., P9/10). For every acoustic modulation frequency (i.e., 4, 20, 40, and 80 Hz), posterior electrodes recorded the highest voltages as depicted by the blue color in the whole-head voltage topographic maps (see Figure 3). In line with this, Figure 5 shows that CP5/6, TP, P, PO, and O electrodes, located on the back of the head, showed the largest response amplitudes. When we ranked the electrode responses by their SNR (highest to lowest), these electrodes were, indeed, ranked highest. Although electrode pairs P1/2 and P3/4 showed a high neural response sensitivity, they were excluded from the selection. Due to their location close toward the midline, these electrodes might have recorded neural responses that were generated in the opposite hemisphere, which, in turn, would bias outcomes concerning hemispheric lateralized activity. Taken together, eight electrode pairs were retained for further analyses (see Figure 2). This data-driven electrode selection covered the principal anatomical regions of auditory processing (e.g., superior temporal gyrus).

#### Neural SNR

The SNR was used as a measure of the size of the ASSR. The higher the neural SNR, the higher the degree of synchronized neural activity. The neural SNR was calculated using the following formula:

$$\text{SNR}\left(\text{dB}\right)=10\times \log_{10}\frac{\sum_{i=1}^{8}\left(\frac{P_{{\left(S+N\right)}_{i}}}{P_{N_{i}}}\right)}{8}$$

*P*_*N*_ was the induced EEG noise. It reflected the power of random, non-synchronized neural activity when the participant was presented with the 4, 20, 40, or 80 Hz modulated noise, and was calculated based on 120 bins adjacent to the 4, 20, 40, or 80 Hz response bin, respectively. We used 60 bins below and 60 bins above the modulation frequency bin, corresponding to ± 0.9 Hz (60 × 0.015 Hz). For instance, *P*_*N*_ induced by the 4 Hz modulated noise, was determined by the average spectral power across the bins ranging 3.085–3.985 Hz and 4.015–4.915 Hz. Throughout this article, we will assign the term “EEG noise” to the noise induced by the acoustic modulations.

*P*_*S*+*N*_ reflected the total power of the Fourier component in the modulation frequency bin and combined the power of the synchronized neural response and the EEG noise.

We calculated the 4, 20, 40, and 80 Hz neural SNR per hemisphere by means of log-transformation of the average SNR across the eight electrodes selected in the LH or RH (see Figure 2). In what follows, “neural SNR” and “ASSR” refer to the hemispheric SNR.

#### Hemispheric lateralization

We examined laterality of processing by means of the laterality index (LI). The LI represented the difference between the root mean square (RMS) response amplitude of the electrode selection in the RH and the LH normalized by the sum of the hemispheric response amplitudes. For each participant, we calculated the LI per stimulus condition (e.g., 40 Hz R) by means of the formula below.

$$\text{LI }=\frac{\text{RMS }{\sqrt{{\text{P}}_{\text{S}+\text{N}}}}_{\text{RH}}-\text{RMS }{\sqrt{{\text{P}}_{\text{S}+\text{N}}}}_{\text{LH}}}{\text{RMS }{\sqrt{{\text{P}}_{\text{S}+\text{N}}}}_{\text{RH}}+\text{RMS }{\sqrt{{\text{P}}_{\text{S}+\text{N}}}}_{\text{LH}}}$$

The RMS amplitude reflected the combined amplitude of the synchronized neural response and the EEG noise. In order to avoid lateralization errors, the LI was only calculated when both hemispheres exhibited a significant ASSR (i.e., the neural SNR was ≥ 4.8 dB). When a significant ASSR was detected in both hemispheres, the LI could not be biased by potential differences in the EEG noise across hemispheres, which might cause lateralization to the “wrong” hemisphere. Positive and negative LIs denoted lateralization to the RH and LH, respectively, whereas an LI of 0 indicated symmetrical processing. LIs could take on any value between −1 and +1.

### Statistical analyses

IBM SPSS Statistics 22.0 software (IBM Corp., Armonk, NY, USA, 2013) was used to perform the statistical analyses that are outlined below.

#### Neural SNR

Per age group, the distribution of each of the 24 neural SNRs (4 frequencies × 3 sides of stimulation × 2 hemispheres) was assessed by means of the Shapiro-Wilk test (α = 0.05). Deviations from the normal distribution were only detected for the 4 Hz ASSR in the young (R RH) and middle-aged group (L LH) and for the 20 Hz ASSR in the older group (R LH).

As we did not aim to compare neural SNRs across the four modulation frequencies, a factorial mixed analysis of variance (FM-ANOVA) with the neural SNR (dB) as the dependent variable, was carried out for the 4, 20, 40, and 80 Hz ASSR separately. Hemisphere and side of stimulation were the within-subject variables and age group was the between-subject variable. Mauchly's test examined whether the assumption of sphericity was met. If sphericity was violated, we applied the Greenhouse-Geisser (ε < 0.75) or Huynh-Feldt (ε > 0.75) correction to examine interaction effects with age (Girden, 1992). *Post-hoc* comparisons were based on the conservative Bonferroni procedure. If Levene's test indicated that the assumption of homogeneity of variance was violated, we used the Games-Howell procedure for *post-hoc* testing of the main effect of age.

To control for the violations of normality, we also performed non-parametric FM-ANOVAs based on Aligned Rank Transformation (Wobbrock et al., 2011). Because the results of the non-parametric ANOVAs corresponded to the parametric outcomes, only the parametric results will be reported.

#### Hemispheric lateralization

As explained before, LIs were only calculated when the participant exhibited a significant ASSR in both hemispheres (SNR ≤ 4.8 dB). Consequently, 62, 76, 100, and 67% of the participants were included in the LI analyses of the 4, 20, 40, and 80 Hz ASSR, respectively.

Per age group, the Shapiro-Wilk test assessed the distribution of each of the 12 LIs (4 frequencies × 3 sides of stimulation) (α = 0.05). No deviations from the normal distribution were detected. A series of one-sample *t*-tests was carried out to investigate whether the age group exhibited LIs that were significantly different from zero. To control for multiple testing, the α level was adjusted to 0.01. A significant positive LI indicated a RH dominance, a significant negative LI reflected a LH dominance, and a non-significant LI denoted a symmetrical response pattern.

We examined possible effects of age on the 4, 20, 40, and 80 Hz LI by means of FM-ANOVAs and *post-hoc* testing. The implementation was similar to the analyses regarding the neural SNR.

For the 4 Hz and 80 Hz LI, we also conducted one-sample Wilcoxon Signed Rank Tests and non-parametric FM-ANOVAs based on Aligned Rank Transformation (Wobbrock et al., 2011). Although the assumption of normality was met, these non-parametric analyses were recommended as, for some age groups, < 10 participants were included in the analyses. These small data sets were due to the lack of subjects having a significant ASSR in both hemispheres. Similar results were obtained from the parametric and non-parametric analyses, unless stated otherwise.

#### Potential confounding factors

To assure that differences in the degree and/or lateralization of synchronized neural activity across the three age groups were not mediated by unwanted confounds, two potential confounding factors were investigated in detail, i.e., peripheral hearing and EEG noise.

##### Peripheral hearing

We included exceptional participants as all, even the older adults, had good audiometric thresholds (≤ 25 dB HL) at all octave frequencies from 125 Hz up to and including 4 kHz. Despite this strict inclusion criterion, it seemed that audiometric thresholds were poorer in the older groups (see Figure 1). Also, there had been no criteria regarding audiometric thresholds at 6 and 8 kHz. Elevated thresholds (> 25 dB HL) at 6 and/or 8 kHz were present in a minority of young and middle-aged participants (5 and 15%, respectively), whereas it was observed in the majority of older participants (86%). This high-frequency hearing loss—although moderate—may affect neural temporal processing of the modulated noise centered at 1 kHz. This assumption is corroborated by psychophysical studies showing that high-frequency hearing loss is accompanied by temporal processing disorders in low-frequency regions where hearing thresholds are within the normal range (Feng et al., 2010; Leigh-Paffenroth and Elangovan, 2011).

Since the assumption of normality was met (*p* > 0.05), a one-way ANOVA denoted whether the age groups differed significantly for the pure tone average across all audiometric frequencies (PTA_125-8000*Hz*_). Semi-partial correlations were determined between the PTA_125-8000*Hz*_ and the neural SNR/LI for which a significant effect of age was observed. These correlations quantified the relationship between peripheral hearing status and the neural SNR/LI while controlling for the effect of age on the PTA. Non-significant semi-partial correlations indicated that differences in peripheral hearing did not mediate the observed differences in neural SNR and/or LI across the three age groups. Because multiple correlations were tested for significance, an α level of 0.01 was implemented.

##### EEG noise

Age-related differences in the amount of EEG noise (i.e., random neural activity, induced by the modulated sound), may affect the neural SNR. More specifically, lower noise amplitudes may cause larger ASSRs and vice versa (see formula in Section ASSR Evaluation - Neural SNR).

Noise amplitudes were calculated by taking the square root of *P*_*N*_ (see Section ASSR Evaluation - Neural SNR). The assumption of normality was only met for the 4 Hz noise amplitude (*p* > 0.05). To investigate age-related differences in the EEG noise, we implemented parametric and non-parametric FM-ANOVAs with the noise amplitude (μV) as the dependent variable. The analyses were similar to the ones regarding the neural SNR. Similar results were obtained from the parametric and non-parametric analyses, unless stated otherwise. We determined semi-partial correlations between the noise amplitude and the neural SNR to verify whether both variables were related when the effect of age on the noise amplitude was controlled for. Non-significant correlations implied that differences in the amount of EEG noise did not contribute to the differences in neural SNR that were detected across the age groups. As multiple correlations were tested for significance, the α level was adjusted to 0.01.

## Results

### Neural SNR

**Figure 6:**
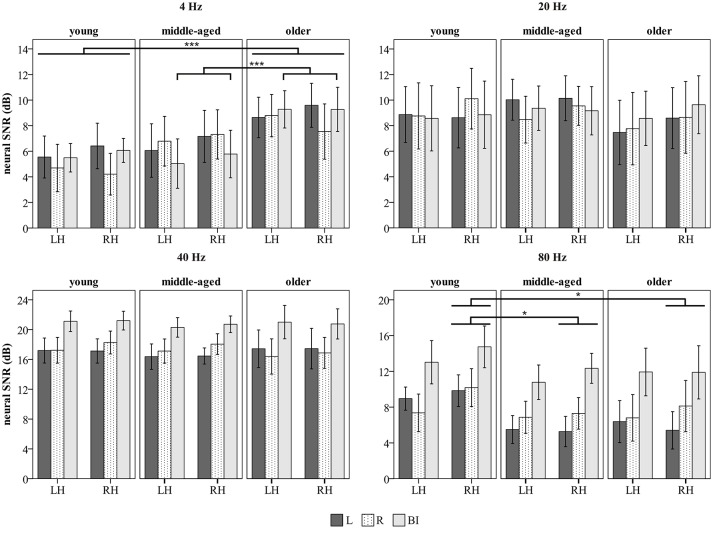
**Neural SNR**. Overview of the neural SNRs (dB) in response to the 4, 20, 40, and 80 Hz acoustic modulations. Age groups (young, middle-aged, older) are displayed per column. The bars are clustered per hemisphere (LH, RH) and represent the average neural SNR per side of stimulation, i.e., L (dark fill), R (dotted fill), and BI (gray fill). Error bars indicate the 95% CI. ^*^p ≤ 0.05; ^***^p ≤ 0.001.

Figure 6 gives an overview of the neural SNRs. A significant main effect of age was identified for the 4 Hz neural SNR [*F*_(2, 50)_ = 5.89, *p* = 0.005]. Older participants had a significantly higher neural SNR than young persons (*mean difference (MD)* = 3.45 dB, *p* = 0.001). Although not statistically significant, a larger 4 Hz response was observed in older compared to middle-aged adults as well (*MD* = 2.49 dB, *p* = 0.056). Young and middle-aged participants had similar 4 Hz neural SNRs (*MD* = 0.95 dB, *p* = 0.59). There was no significant interaction between age group and hemisphere [*F*_(2, 50)_ = 1.99, *p* = 0.147], but there was a significant interaction between age group and side of stimulation [*F*_(4, 100)_ = 2.66, *p* = 0.037]. Bonferroni *post-hoc* testing indicated that older listeners exhibited a higher 4 Hz neural SNR than young subjects, irrespective of the side of stimulation (L: *MD* = 3.13 dB, *p* = 0.052; R: *MD* = 3.71 dB, *p* = 0.017; BI: *MD* = 3.49 dB, *p* = 0.005). Relative to the middle-aged group, the 4 Hz response in the older group was larger for BI stimulus presentation only (*MD* = 3.87 dB, *p* = 0.001).

For the 20 and 40 Hz neural SNR, neither a main effect of age nor an interaction effect was observed.

The FM-ANOVA concerning the 80 Hz neural SNR revealed a significant main effect of age [*F*_(2, 50)_ = 3.22, *p* = 0.049]. *Post-hoc*, a larger 80 Hz ASSR was observed in the young compared to the middle-aged group, but this difference was not significant (*MD* = 2.67 dB, *p* = 0.061). Young and older participants exhibited similar 80 Hz neural SNRs (*MD* = 2.26 dB, *p* = 0.213). The interaction between age group and hemisphere was significant [*F*_(2, 50)_ = 3.26, *p* = 0.047]. In the LH, the 80 Hz response was equally large across the three age groups. In the RH, the neural SNR was higher in young than middle-aged participants (*MD* = 3.28 dB, *p* = 0.022) and there was a clear tendency for larger 80 Hz responses in young compared to older adults as well (*MD* = 3.11 dB, *p* = 0.058). There was no interaction between age group and side of stimulation [*F*_(4, 100)_ = 2.04, *p* = 0.094].

### Hemispheric lateralization

**Figure 7:**
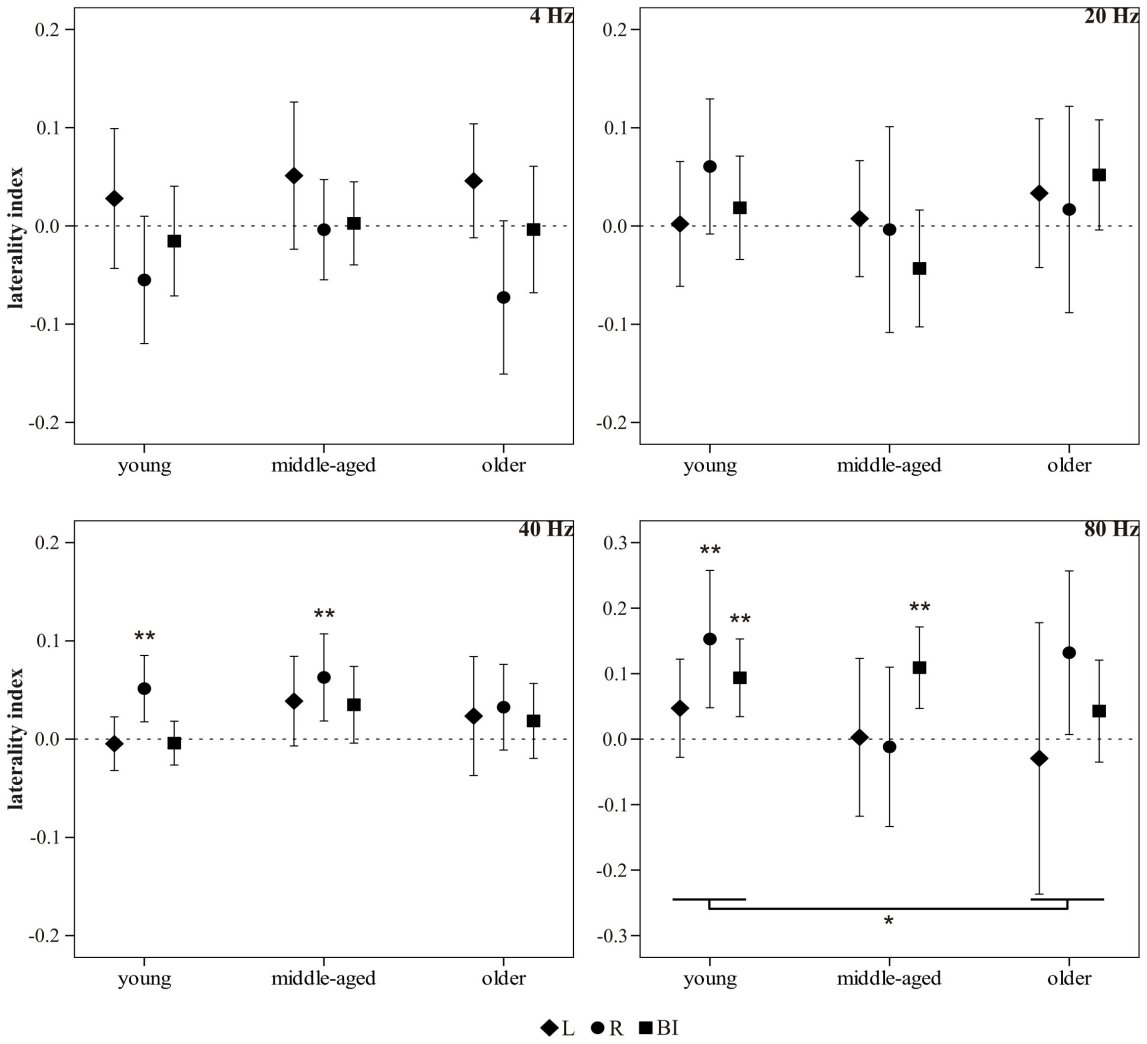
**Laterality index**. Overview of the LIs for the 4, 20, 40, and 80 Hz acoustic modulations. The markers are clustered per age group (young, middle-aged, older) and represent the average LI per side of stimulation, i.e., L (triangles), R (circles), and BI (squares). Error bars indicate the 95% CI. ^*^p ≤ 0.05; ^**^p ≤ 0.01.

The LIs are displayed in Figure 7. In the young group, a significant RH dominance was demonstrated for three stimulus conditions, i.e., 40 Hz R [*t*_(18)_ = 3.20, *p* = 0.005, *N* = 19], 80 Hz R [*t*_(13)_ = 3.15, *p* = 0.008, *N* = 14], and 80 Hz BI [*t*_(16)_ = 3.35, *p* = 0.004, *N* = 17]. In the middle-aged group, a RH asymmetry was detected for two stimulus conditions, i.e., 40 Hz R [*t*_(19)_ = 2.96, *p* = 0.008, *N* = 20] and 80 Hz BI [*t*_(18)_ = 3.69, *p* = 0.002, *N* = 19]. In the older group, the *t*-tests identified no significant LIs.

For the 4, 20, and 40 Hz LI, neither a main effect of age nor an interaction effect was indicated by the FM-ANOVA, whereas for the 80 Hz LI (*N*_young_ = 13, *N*_middle-*aged*_ = 6, *N*_older_ = 4), a significant main effect of age was observed [*F*_(2, 20)_ = 4.14, *p* = 0.031]. Bonferroni *post-hoc* testing showed a tendency for the 80 Hz LI being more positive in the young compared to the older group (*MD* = 0.10, *p* = 0.052), while there was no significant difference between the young and middle-aged group, nor between the middle-aged and older group. There was no interaction between age group and side of stimulation [*F*_(4, 40)_ = 1.64, *p* = 0.184]. The non-parametric FM-ANOVA confirmed the main effect of age for the 80 Hz LI [*F*_(2, 20)_ = 3.58, *p* = 0.047], and indicated a significant difference between the young and older group (*MD* = 22.05, *p* = 0.044).

### Potential confounding factors: peripheral hearing and EEG noise

Descriptive statistics of the PTA and EEG noise are provided in Table 2.

**Table 2:** **Descriptive statistics PTA and EEG noise**.

	**Young**	**Middle-aged**	**Older**
**PTA_125-8000 Hz_**			
Mean ± SD	3.7 ± 3.6	8.8 ± 4.1	17.7 ± 4.7
Minimum; maximum	−3.2; 11.9	3.1; 20.0	10.9; 28.1
**4 Hz EEG NOISE**			
Mean ± SD	0.189 ± 0.026	0.146 ± 0.021	0.158 ± 0.036
Minimum; maximum	0.152; 0.240	0.102; 0.180	0.108; 0.216
**80 Hz EEG NOISE**			
Mean ± SD	0.019 ± 0.006	0.024 ± 0.008	0.027 ± 0.011
Minimum; maximum	0.010; 0.033	0.015; 0.038	0.017; 0.058

Age group's mean ± SD, minimum, and maximum of the PTA_125-8000Hz_ (dB HL), 4 Hz EEG noise (μV), and 80 Hz EEG noise (μV).

Although all participants had audiometric thresholds within clinically normal limits up to and including 4 kHz, a significant effect of age was observed for the PTA_125-8000*Hz*_ [*F*_(2, 50)_ = 47.73, *p* < 0.001]. Young participants had better audiometric thresholds compared to middle-aged (*MD* = 5.1 dB HL, *p* = 0.001) and older adults (*MD* = 14.0 dB HL, *p* < 0.001), and middle-aged persons had better thresholds than older participants (*MD* = 8.9 dB HL, *p* < 0.001).

For the EEG noise, we investigated possible effects of age on the 4 and 80 Hz noise amplitudes as significant group differences in neural SNR and LI involved the 4 and 80 Hz ASSR. A main effect of age was revealed for both the 4 and 80 Hz EEG noise [4 Hz: *F*_(2, 50)_ = 12.85, *p* < 0.001; 80 Hz: *F*_(2, 50)_ = 3.96, *p* = 0.025]. No interaction effects with hemisphere or side of stimulation were identified. *Post-hoc* testing showed that the amplitude of the EEG noise at 4 Hz was equally large for the middle-aged and older group (*MD* = 0.01 μV, *p* = 0.477), and that the amplitude of the young group was significantly larger (young vs. middle-aged: *MD* = 0.04 μV, *p* < 0.001; young vs. older: *MD* = 0.03 μV, *p* = 0.031). For the 80 Hz EEG noise, a significant difference was identified between young and older adults only, with the noise amplitude being larger in the older group (*MD* = 0.008 μV, *p* = 0.031).

Semi-partial correlational analyses were carried out (α = 0.01) to investigate whether these age-related differences in PTA and EEG noise mediated the significant group effects regarding the 4 Hz neural SNR, 80 Hz neural SNR, or 80 Hz LI. The correlation coefficients are outlined in Table 3.

**Table 3:** **Semi-partial correlation coefficients**.

	**Overall**	**LH**	**RH**	**L**	**R**	**BI**
PTA – 4 Hz ASSR	0.24	0.31	0.18	0.21	0.14	0.33
PTA – 80 Hz ASSR	0.09	0.17	0.01	0.13	0.16	0.05
PTA – 80 Hz LI	–	–	–	−0.07	−0.01	−0.22
EEG noise – 4 Hz ASSR	−0.08	−0.05	−0.11	0.00	−0.23	−0.05
EEG noise – 80 Hz ASSR	0.09	0.05	0.06	0.14	0.00	0.03

Semi-partial correlation coefficients concerning the neural SNR (ASSR) were calculated irrespective of hemisphere and side of stimulation (overall), per hemisphere (LH, RH), and per side of stimulation (L, R, BI). For the LI, semi-partial correlation coefficients were calculated per side of stimulation.

For the PTA, no significant semi-partial correlation coefficients were detected. Although, in the LH and for BI stimulus presentation, there was a tendency for the 4 Hz ASSR being larger if the PTA was higher (*p* = 0.018 and *p* = 0.012, respectively). For the EEG noise, none of the correlation coefficients reached significance (all *p* > 0.08).

## Discussion

In order to gain more insight into the underlying mechanisms of age-related speech perception difficulties, we investigated whether aging affects the degree and/or lateralization of synchronized theta, beta, and gamma activity in response to speech-related acoustic modulations. To that end, we recorded 4, 20, 40, and 80 Hz ASSRs in stringently selected young, middle-aged, and older adults. All participants had clinically normal hearing and there were no indications of (mild) cognitive impairment. This is exceptional, in particular for the older adults. Our data yield novel insights into the synchronized activity of theta and of high gamma oscillations in the aging auditory system.

### More synchronized activity of theta oscillations at an older age

Older participants exhibited increased theta synchronization as reflected by larger 4 Hz ASSRs relative to young and middle-aged persons who showed similar neural responses. For the 4 Hz response in the LH and for BI stimulus presentation, we detected weak PTA-neural SNR correlations, suggesting that the neural SNR increases when the PTA increases. Nonetheless, we do not believe that the increased theta synchronization in older persons is due to their slightly elevated hearing thresholds. The 4 Hz ASSR is increased in the older group, irrespective of hemisphere and side of stimulation, but the correlational analyses point toward a poor PTA-neural SNR link for one hemisphere and for one side of stimulation only. Also, if the PTA were the driving force behind increased theta synchronization, the middle-aged group would presumably exhibit a larger 4 Hz ASSR as well since their PTA is significantly higher than the PTA of the young group. Taken together, we believe that the increased synchronized theta activity in older persons is not mediated by their peripheral hearing status.

Our data are in line with those of Tlumak et al. (2015) who showed that 5 Hz ASSRs were increased in normal-hearing older adults compared to young listeners.

Age-related declines in neural inhibition may explain this effect of age. It has been demonstrated that the 4 Hz ASSR has its main neural generator in the auditory cortex (Giraud et al., 2000; Herdman et al., 2002). A large body of research has shown that there is an age-related decrease in neural inhibitory activity, mediated by the neurotransmitter GABA, at the level of the cortex. This decrease originates from an age-related down-regulation in GABA synthesis and release, and from changes in the composition of GABA receptors (e.g., Caspary et al., 2008, 2013). Since GABAergic inhibition is a major determinant of neuronal excitability (Jacob et al., 2008; Gray et al., 2014), it seems plausible that these age-related neurochemical changes mediate the increased synchronized activity of theta oscillations in older persons. In addition, there are age-related anatomical changes that relate to decreased neural inhibition. A reduction in gray and white matter volume has been detected in various regions of the aging brain, including the frontal and temporal-auditory lobes (Bartzokis et al., 2001; Walhovd et al., 2005). These anatomical changes likely reflect an elimination of active synapses, and as such, a loss of reciprocal connections between the frontal and auditory cortices. Importantly, these connections seem to be indispensable for accurate temporal auditory processing (Griffiths et al., 2000). This is likely explained by frontal lobe-mediated inhibition onto neurons in the auditory cortex (Weisser et al., 2001). Age-related degradations of these inhibitory connections may give rise to increased responsiveness of theta oscillations in the auditory cortex at an older age.

Why these age-related inhibitory changes affect the 4 Hz ASSR, while the other cortical response of our study, the 20 Hz ASSR, appears to remain unaffected, might be explained by both ASSRs having different neural generators, each tuned to a particular frequency range of acoustic modulations. A number of studies have demonstrated that the 4 and 20 Hz ASSR, indeed, originate from distinct cortical areas, the secondary auditory cortex and medial geniculate body, respectively (e.g., Preuss and Müller-Preuss, 1990; Giraud et al., 2000). Moreover, the assumption that these different cortical regions are tuned to different frequency ranges, is supported by the modulation filterbank model of Dau et al. (1997) which accurately predicts behavioral outcomes on amplitude modulation detection, based on this assumption.

### Less synchronized activity of high gamma oscillations at an older age

80 Hz ASSRs were smaller in the middle-aged and older groups compared to the young group. We conclude that the decline in synchronized activity of high gamma oscillations is related to age since minor differences in peripheral hearing and EEG noise appear not to be involved.

A number of previous ASSR studies have also demonstrated that neural synchronization to high-frequency acoustic modulations is affected with age (Dimitrijevic et al., 2004; Purcell et al., 2004; Leigh-Paffenroth and Fowler, 2006; Grose et al., 2009; Parthasarathy et al., 2010).

This ASSR outcome is in line with ERP studies indicating an age-related reduction in neural synchronization at the level of the brainstem (e.g., Anderson et al., 2012; Bidelman et al., 2014; Mamo et al., 2016), presumably because high-frequency ASSRs (≥ 80 Hz) are mainly generated by cochlear nuclei in the brainstem (Giraud et al., 2000; Herdman et al., 2002). A major brainstem source of the 80 Hz ASSR is also evidenced by studies showing that the 80 Hz ASSR is not affected by the state of arousal (Cohen et al., 1991; Purcell et al., 2004).

In the aging auditory brainstem, reduced levels of the inhibitory neurotransmitter GABA have been observed (e.g., Caspary et al., 2008). Interestingly, older brainstems seem to exhibit a compensation for this reduced GABA release. It has been demonstrated that there is a significant age-related increase in GABA receptor subunits that boost the influx of GABA (Milbrandt et al., 1997; Caspary et al., 1999). These age-related GABAergic changes result in an up-regulation of GABAergic inhibition at brainstem level, which is in line with our results, indicating smaller 80 Hz ASSRs in aging persons.

An alternative explanation for the age-related decrease in gamma synchronization involves anatomical changes as has been indicated in the brainstem of aging animals. With advancing age, there is a substantial reduction in afferent neural projections onto the inferior colliculus, possibly a result of neuronal loss in the cochlear nucleus (Frisina, 2001; Frisina and Walton, 2006). This deafferentation appears to be associated with temporal processing deficiencies in older subjects (e.g., Ison et al., 1998).

Age-related deviances in neural recovery from excitation are in line with our results as well. Neural recovery in auditory brainstem centers is prolonged in older compared to young subjects and this age effect is most salient when acoustic events are separated by ≤ 16 ms (Walton et al., 1998, 1999). By virtue of these findings, we suggest that, for our older groups, the interval between successive 80 Hz modulation cycles (~12 ms) was too short for the high gamma oscillatory neurons to recover from the initial period of excitation. This resulted in poor encoding of the modulations as reflected by the smaller 80 Hz ASSRs.

### Symmetrical synchronized activity of high gamma oscillations at an older age

Our results indicate that processing of high gamma range modulations is significantly lateralized to the RH in young persons, whereas this RH dominance is not exhibited by older adults. Based on the applied selection criteria and the outcomes of the correlational analyses concerning peripheral hearing, we conclude that the absence of a RH dominance at an older age can be attributed to aging.

The observed RH dominance in our young group is consistent with findings from MEG studies. Schoonhoven et al. (2003) investigated synchronized brain activity in response to 1000 Hz pure tones modulated at 80 Hz. Their participants (18–35 years) showed a bilateral activation of the parietal/temporal lobe with a higher degree of synchronized activity over the RH than over the LH. In line with this, Hertrich et al. (2004) presented sweeps at repetition rates of 67 and 111 Hz diotically to both ears in a group of young adults. They demonstrated that the synchronized activity was more pronounced in the RH compared to the LH.

To the best of our knowledge, only one other study has investigated effects of age on hemispheric asymmetry of low-level auditory processing. Bellis et al. (2000) examined the cortical P1-N1 complex in response to synthetic speech syllables in normal-hearing young and older adults (20–25 years and > 55 years, respectively). They reported larger P1-N1 responses over the left than over the right temporal lobe in the young group, whereas the older subjects showed a symmetrical response pattern. These results correspond to ours as the hemispheric lateralization exhibited by young persons was not observed in the older group.

Symmetrical response patterns at an older age can be associated with age-related callosal dysfunctions. It is suggested that a hemispheric dominance arises from competitive interhemispheric inhibition, partly mediated by interhemispheric transfer through the corpus callosum (Cook, 1984; Chiarello and Maxfield, 1996). Imaging studies have demonstrated corpus callosum atrophy and demyelination with aging (Janowsky et al., 1996; Silver et al., 1997). These morphological changes may affect interhemispheric conduction efficiency, which, in turn, affects the interhemispheric inhibitory processes that drive hemispheric lateralized activity. This hypothesis is supported by age-related performance differences on tasks that rely on interhemispheric integrity. In dichotic speech perception tasks, older adults show a pronounced left ear deficit (Jerger and Jordan, 1992; Jerger et al., 1994; Bellis and Wilber, 2001), which is in line with an age-related decline in interhemispheric transfer efficiency via the corpus callosum. After all, in contrast to the right ear input that is presumed to access the left hemisphere directly, the left ear input must transverse the corpus callosum to reach the left hemisphere, which is known to be the dominant hemisphere for processing of speech.

An alternative explanation for age-related differences in hemispheric asymmetry has been offered by Chen et al. (2013). In this study, young (18–27 years) and older adults (55–75 years) with clinically normal hearing underwent magnetic resonance spectroscopy scans in order to investigate hemispheric contents of glutamate and GABA, two neurotransmitters that mediate excitatory and inhibitory processes. In the young group, they detected a RH asymmetry for glutamate and a LH asymmetry for GABA. These asymmetries were only minimally exhibited by the normal-hearing older group. The researchers suggested that this age-related reduction in asymmetrical distribution of regulating neurotransmitters might underlie the decline in functional lateralization for linguistic and cognitive processing in aging persons (Bellis et al., 2000; Greenwald and Jerger, 2001; Berlingeri et al., 2013).

Another plausible explanation involves age-related changes in brain volume. N-acetylaspartate (NAA) is a cortical metabolite that is almost exclusively present in neurons, neuronal dendrites, and axons. As such, the NAA percentage is used to evaluate neuronal damage or loss. Spectroscopic investigations have demonstrated that NAA concentrations in the temporal-auditory brain regions decrease with advancing age, reflecting neuronal dysfunction and/or loss in the aging brain (Angelie et al., 2001; Chen et al., 2013). This NAA observation is consistent with imaging studies demonstrating an age-related reduction in brain volume (e.g., Matsumae et al., 1996; Lim and Spielman, 1997). By virtue of the remarkable plasticity of the human brain (e.g., Gold and Bajo, 2014), we assume that the neuronal dysfunction/loss at an older age, evokes functional reorganizations in the aging brain, which, amongst other outcomes, result in more symmetrical patterns of neural processing.

### The interplay between neural synchronization and speech perception

Whether the observed age-related differences in synchronization of theta and high gamma oscillations to speech-related acoustic modulations are associated with the well-known speech perception difficulties in aging persons is an intriguing issue. There is evidence in literature that such changes in neural synchronization are indeed related to speech perception deficits.

In a recent study, EEG recordings in response to the speech sound /da/ were collected from children with and without speech intelligibility problems (Gilley et al., 2016). A significant dissimilarity between both groups was detected in the spectral magnitude of the theta peak. More specifically, a larger theta peak, reflecting more synchronized theta activity, was observed in children with speech perception difficulties. The assumption that an increased 4 Hz ASSR may be a neural marker for speech perception difficulties, is in line with the predictive coding theory (Friston, 2005; Sohoglu et al., 2012). This theory attributes increased cortical neural responsiveness to the presence of a larger predictive error component in the neural response. The error component signals that the auditory input is not properly encoded. Stated otherwise, neural responses increase when the error component increases, and, as such, larger ASSRs indicate poorer encoding of the acoustic signal. Taken together, increased theta synchronization in our older group is suggested to reflect poorer encoding of theta range modulations. Accurate processing of these low-frequency modulations, however, is crucial for parsing connected speech into meaningful, syllable-sized chunks, leading to accurate speech perception (e.g., Doelling et al., 2014).

With regard to the 80 Hz ASSR, previous studies have demonstrated that smaller high gamma responses are associated with poorer speech perception performance. For instance, Leigh-Paffenroth and Fowler (2006) detected significant correlations between the synchronization capability of gamma range oscillations and word recognition scores within a group of normal-hearing young and older participants. The number of significant 90 Hz ASSRs decreased when word recognition performance decreased. Along similar lines, Dimitrijevic et al. (2001, 2004) observed significant positive correlations between word recognition scores and the number of significant ASSRs to pure tones that were modulated at several high-frequency rates (78–100 Hz).

Abnormal lateralization patterns appear to have adverse effects on speech processing as well. In the study of Bellis et al. (2000), young adults demonstrated an asymmetrical pattern for the P1-N1 response, elicited by synthetic speech syllables, whereas normal-hearing older persons showed a symmetrical response pattern. This reduction in hemispheric asymmetry was accompanied by a reduced ability to discriminate speech syllables in the older group. In line with this, Marvel et al. (1992) observed systematic differences in the hemispheric distribution of auditory evoked potentials between an older group with good speech understanding and older persons with poor speech understanding. Moreover, it has been suggested that dyslexia, a linguistic disorder characterized by phonological processing problems, arises from an altered hemispheric asymmetry of neural synchronization to speech-related acoustic modulations (Goswami, 2011; Hämäläinen et al., 2012).

In order to be able to draw conclusions regarding the functional consequences of increased theta synchronization, decreased high gamma synchronization, and symmetrical high gamma response patterns with advancing age, we will investigate the speech perception abilities of our young, middle-aged, and older participants in a follow-up study. Based on the above-mentioned research findings, it seems plausible that the observed age-related changes in the central auditory system contribute to speech perception problems in aging persons. Therefore, we hypothesize that speech perception performance will be worse in our older compared to our young participants.

## Conclusions

This study yields novel insights into how synchronized activity of theta oscillations and synchronization of high gamma oscillations in the central auditory system change with age. Our results indicate that, at an older age, synchronized activity of theta oscillations is increased, whereas synchronization of high gamma oscillations is decreased. Also, in contrast to young individuals who exhibit a RH dominance for processing of high gamma range modulations, older listeners show a symmetrical response pattern. These age-related changes in neural synchronization may very well underlie the impaired speech perception in aging persons. Further investigation is warranted to endorse this assumption, which, in turn, may impact clinical decisions regarding auditory rehabilitation and may lead to novel training strategies to prevent older persons from missing out on conversations.

## Author contributions

All authors listed, have made substantial, direct and intellectual contribution to the work, and approved it for publication.

### Conflict of interest statement

The authors declare that the research was conducted in the absence of any commercial or financial relationships that could be construed as a potential conflict of interest.

## Abbreviations

ASSR: auditory steady-state response
RH: right hemisphere
LH: left hemisphere
L: left ear
R: right ear
BI, bilaterally: diotically to both ears
FFT: Fast Fourier Transformation
SNR: signal-to-noise ratio
LI: laterality index
RMS: root mean square
(FM-)ANOVA: (factorial mixed) analysis of variance
PTA: pure tone average
MD: mean difference.
